# Sport Related Sudden Death: The Importance of Primary and Secondary Prevention

**DOI:** 10.3390/jcm11164683

**Published:** 2022-08-11

**Authors:** Antonio Gianluca Robles, Zefferino Palamà, Martina Nesti, Roberto Michele Tunzi, Pietro Delise, Elena Cavarretta, Maria Penco, Silvio Romano, Luigi Sciarra

**Affiliations:** 1 Department of Life, Health and Environmental Sciences, University of L’Aquila, 67100 L’Aquila, Italy; 2 Cardiovascular and Neurological Department, Ospedale San Donato, 52100 Arezzo, Italy; 3 GVM Care e Research, Anthea Hospital, 70124 Bari, Italy; 4 Division of Cardiology, Hospital ‘P. Pederzoli’, 37019 Peschiera del Garda, Italy; 5 Department of Medical-Surgical Sciences and Biotechnologies, Sapienza University of Rome, 04100 Latina, Italy; 6 Mediterranea Cardiocentro, 80122 Naples, Italy

**Keywords:** cardiac arrest, defibrillation, resuscitation, AED, sport pre-participation screening

## Abstract

Sports are a double-edged sword. On the one hand, cardiovascular benefits from sports activity are well-known, and on the other hand, sports may increase the risk of sudden cardiac death (SCD) in subjects with known or unknown cardiovascular diseases. SCD is rare but has a very strong emotional issue. There are many examples involving famous professional athletes, but this is only scratching the surface of a widespread phenomenon that also involves amateur athletes. The importance of safely performing physical activity appears clear in both professional and amateur athletes. In particular, the former undergo a pre-participation screening for SCD primary prevention with different recommendations in each country. On the other hand, a medical examination is not mandatory for non-professional athletes and, therefore, for people who practice sports as an amateur. Widespread distribution of automatic external defibrillators and people trained for cardiopulmonary resuscitation are necessary to promote secondary prevention of SCD. We briefly report a case series of athletes with aborted SCD during sports activity in order to underline and discuss in this review the previously highlighted issues.

## 1. Introduction

Each year, sudden cardiac death (SCD) affects 1–3/100.000 competitive athletes aged <35 years worldwide [1,2,3,4]. The most frequent causes of sports-related SCD are hypertrophic cardiomyopathy (United States) and arrhythmogenic cardiomyopathy (Italy) in <35-year-old subjects, coronary artery disease in older ones, and channelopathies (e.g., LQT1-2 and catecholaminergic polymorphic ventricular tachycardia) in children [5,6,7,8,9].

The patients are often asymptomatic, with excellent physical performance, and the first manifestation of the disease is SCD. A screening electrocardiogram (ECG) can be a useful and simple tool to unmask these pathologies. Italian (COCIS), European, and American guidelines provide recommendations on sports eligibility in the case of cardiovascular anomalies [10,11,12,13,14]. They are roughly similar, even if the Italian ones appear more restrictive. However, the sports-related SCD is rare, so the cost-effectiveness of mass screening is doubtful, it is not applied to all categories of athletes, and it has differences among countries. For example, in Italy, ECG screening is mandatory for professional athletes and recently also for non-professionals; conversely, it is not mandatory in the United States [15,16]. For these reasons, the pre-participation screening is a good starting point, but it is not the only solution to the problem, and therefore we must try to make sports activities as safe as possible. The presence of automatic external defibrillators (AEDs) in sports centers and during competitions should be forced [17,18]. However, sports activity can be practiced by anyone even in the most remote and isolated places. What can we do to avoid SCD outside a *“cardioprotected”* site? The acquisition of outdoor AEDs and increasing cardiopulmonary resuscitation (CPR) training for all populations should be our targets to reduce SCD during sports activities.

In the following, a case series of four aborted sports-related SCDs is presented.

## 2. Case 1

**Figure 1:**
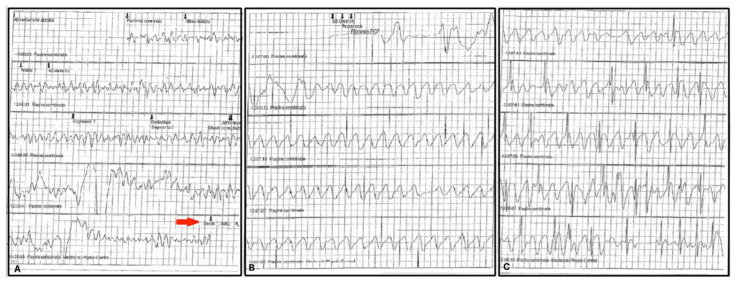
Lifepak-500 (Medtronic, Minneapolis, MI, USA) registered traces during CPR maneuvers. (**Panel A**) ventricular fibrillation recognition and subsequent 360J shock discharge (red arrow) by AED. (**Panel B** and **C**) post-shock normal rhythm restoration and artifacts from chest compressions.

An 18-year-old Caucasian male with no known family history (adopted at an early age from a foreign country) suffered from cardiac arrest during a competitive soccer match in an Italian regional league. CPR was immediately started by the soccer team’s coach. The medical ambulance present for the event was immediately activated. Ventricular fibrillation was recognized after rhythm analysis by AED and a 360 J discharge restored sinus rhythm (Figure 1). In the hospital, echocardiography revealed abnormal hypertrophy, especially of the interventricular septum (19 mm), and coronary angiography showed a small muscle bridge at the left anterior descending coronary middle tract. Cardiovascular magnetic resonance (CMR) imaging confirmed asymmetrical septal hypertrophy and showed focal intramyocardial late gadolinium enhancement areas spread in the left ventricle (expression of myocardial fibrosis). A subcutaneous implantable cardioverter defibrillator (S-ICD—Boston Scientific, Marlborough, MA, USA) was implanted, and a few months later, a mutation of the MYH7 gene-encoding β-cardiac myosin heavy chain was revealed by genetic testing.

**Figure 2:**
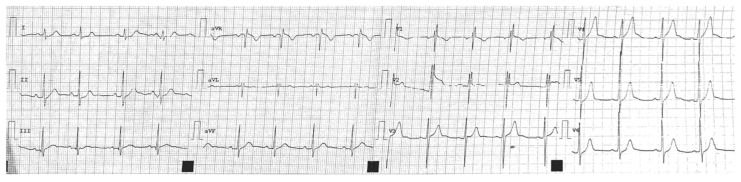
Resting ECG performed during sports screening. We can see Sokolow–Lyon Criteria for left ventricular hypertrophy (S wave in V1 + R wave in V5 or V6 ≥ 36 mm in male) and pathological J-ST depression in V6. There is possibly a J-ST depression in leads I and II, but we cannot be sure because of the quality of the resting ECG available. Finally, there is partial right bundle branch block (rSR’ pattern in V1–V2), but it is not a pathological finding.

The soccer player, according to the Italian sports medicine protocol [9], was screened 10 months earlier, with a rest ECG and with a step test. The retrospective analysis of this ECG revealed the presence of the voltage ventricular hypertrophy criteria (Figure 2), in the absence of other clear malignant criteria (T-wave inversion in inferior–lateral leads, pathological Q waves, and left axis deviation) [11]. There was only a mild J-ST depression in lead V6. For this reason, and because of the absence of family history, the diagnostic flowchart was stopped.

Probably, the presence of this mild J-ST depression in association with only voltage criteria for left ventricle hypertrophy in a young non-professional athlete practicing a low-intensity sport [12] should have suggested a more accurate diagnostic screening with an echocardiogram.

## 3. Case 2

**Figure 3:**
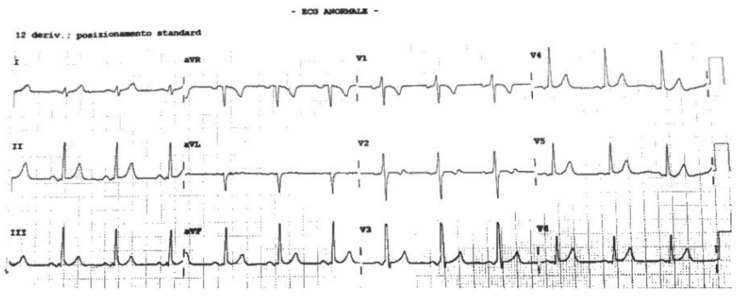
Resting ECG performed during non-competitive-sports screening. A short PQ-interval and a mild delta wave can be observed.

**Figure 4:**
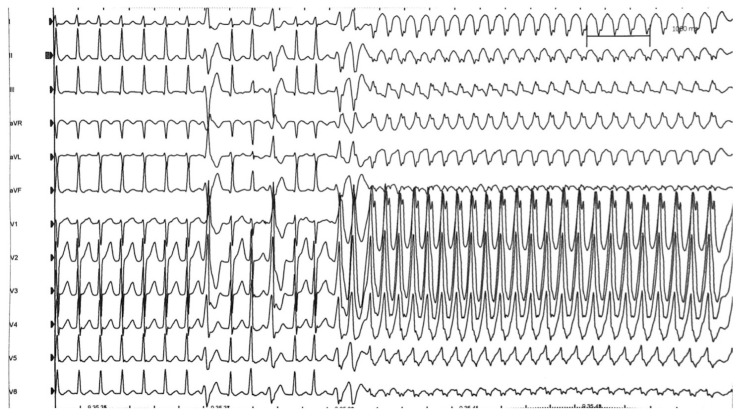
Electrophysiological Study: orthodromic atrioventricular reentrant tachycardia degenerating into atrial fibrillation with a very high ventricular rate response along a left accessory pathway.

**Figure 5:**
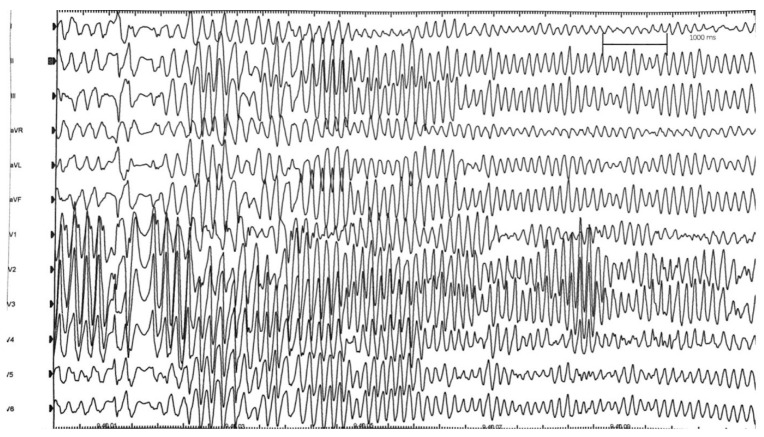
Electrophysiological Study: atrial fibrillation with a very high ventricular rate response along a left accessory pathway degenerating into ventricular fibrillation.

**Figure 6:**
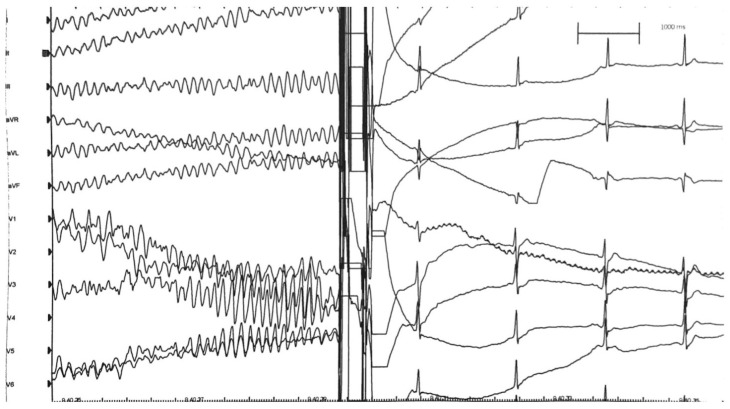
Electrophysiological Study: ventricular fibrillation interruption by external DC shock.

A 17-year-old Caucasian male with no SCD family history suffered from a cardiac arrest during a non-competitive basketball match. The basketball player, according to the Italian sports medicine protocol in non-competitive athletes [10], was screened 4 months earlier, with only a rest ECG (Figure 3), without reporting anomalies. No syncope or tachycardia episodes were reported in his clinical history. The patient was promptly resuscitated with an effective AED discharge (200 J). In the hospital, a carefully 12-lead rest ECG analysis demonstrated short PR duration and the presence of delta wave. The electrophysiological study demonstrated an inducibility of an orthodromic atrioventricular reentrant tachycardia that soon degenerated into atrial fibrillation, with a very high ventricular response along a left accessory pathway (Figure 4), and this, in turn, rapidly degenerated into ventricular fibrillation (Figure 5) and then was interrupted with DC shock (Figure 6). The accessory pathway was effectively treated by radiofrequency ablation. Wolf–Parkinson–White patients with an ECG of difficult interpretation are common. A normal or at the lower limit (120–130 ms) PR interval may occur due to the presence of a long Kent bundle on the left side with delayed conduction [19]. A more careful ECG analysis during the screening should have made the suspicion of pre-excitation because of the short PR interval and the delta wave slurring [20].

## 4. Case 3

**Figure 7:**
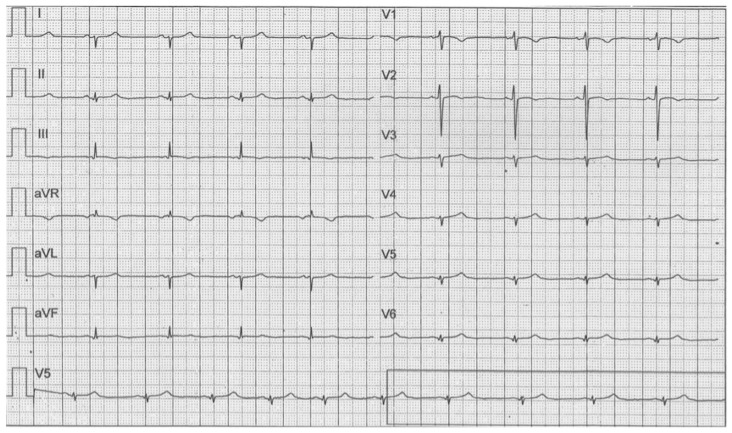
Resting ECG of 29-year-old female swimming teacher, showing right axis deviation of QRS and diffuse low voltages.

A 29-year-old female swimming teacher regularly underwent pre-participation screening. She had always been asymptomatic and in good health. Afterward, when she was swimming, she experienced an *in-water* cardiac arrest. She was soon rescued: CPR was promptly started by her coach colleague, and soon she was defibrillated by the AED available. After this episode, she was admitted to the hospital, where she underwent a full cardiological evaluation. In particular, the coronary angiography and echocardiography showed no anomalies, but the ECG (Figure 7) showed diffuse low voltages and right axis deviation that were not present at previous ECGs. In addition, she underwent cardiac magnetic resonance, which showed the presence of a large non-ischemic left ventricular scar in absence of edema. It was compatible with previous myocarditis with an asymptomatic course during the acute phase. It is possible that the inflammatory process involved the myocardium after the last sports visit because older ECGs did not show these pathological features. At the end of the diagnostic workup, the young patient had an S-ICD implanted for secondary prevention (Boston Scientific, Marlborough, MA, USA).

This case suggests that recurring sports evaluations, including at least one resting ECG, increase the likelihood to unmask unknown pathologies, thus reducing the risk of sports-related SCD.

## 5. Case 4

**Figure 8:**
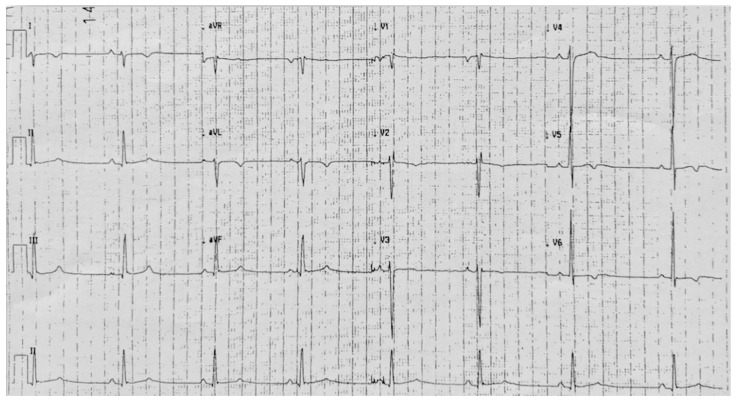
Resting ECG of 30-year-old ex-competitive male soccer player. Right axis deviation, traces of early ventricular repolarization in inferior leads, partial right bundle branch block, and negative T waves in lateral leads (D1-aVL and V5–V6). Negative P-wave terminal force in lead V1 is also present.

A 30-year-old male ex-competitive soccer player had always been in good health. He did not complain of any cardiac symptoms during his career. Afterward, during an amateur soccer match, he experienced an aborted SCD. Effective defibrillation was performed with the aid of the AED available in the sports arena. During the hospitalization, he underwent a resting ECG (Figure 8), showing right axis deviation, traces of early ventricular repolarization in inferior leads, partial right bundle branch block, and negative T waves in lateral leads (D1-aVL and V5–V6). Unfortunately, no previous ECGs for comparison were available. Moreover, he underwent the following diagnostic tests without pathological elements: echocardiography, coronary computed tomography, CMR imaging, IC-drug challenge, screening for main genes of cardiomyopathies (also for Fabry disease) and channelopathies, electrophysiological study (non-inducibility of ventricular arrhythmias with ventricular programmed stimulation performed from two different sites in the right ventricle, and double extra-stimulus with couplings up to ventricular refractoriness or up to 200 msec) and right ventricular endocardial mono/bi-polar voltage 3D mapping (CARTO—Biosense Webster Inc, Irvine, CA, USA). Even a maximal exercise stress test did not elicit ventricular arrhythmias. At the end of the diagnostic workup, the young patient had a transvenous ICD implanted for secondary prevention. The diagnostic suspicion was of Idiopathic Ventricular Fibrillation [21]. The prevention would have not been enough to protect this patient. This case underlines the importance of AED in the sports arena to practice sports safely.

## 6. Discussion

The cases presented invite us to reflect on a topic that is often overlooked: the importance of playing sports safely. Although sports activity generates psycho-physical benefits, in some predisposed subjects, it can involve a risk of SCD [4,22,23,24,25,26,27,28]. Exercise favors supraventricular arrhythmias, but especially ventricular ones, including ventricular fibrillation [29]. Ventricular fibrillation and sudden sports death occur in presence of heart diseases or channelopathies, known or not, which generally allow normal or even high-quality performance, are often asymptomatic but may nevertheless have SCD during sports as a first manifestation [4,5,9]. In the presence of a cardiovascular anomaly, there are Italian (COCIS) [10], European [11,12,13], and American [14] guidelines that provide recommendations on how to behave to be eligible for sport. They are roughly similar. The European and American guidelines indicate good practice for people engaging in physical activity at various levels, not only at the competitive level. In contrast, the COCIS guidelines refer specifically to competitive athletes in various sports, including those with high cardiovascular stress.

The different guidelines have areas of overlap, especially the Italian and European ones, also because the latter’s drafting involved many Italian experts. However, differences also exist: American guidelines tend to be less restrictive and more permissive than the Italians ones, for example. All guidelines are not law, but they contain somewhat flexible recommendations. Practically speaking, in the case of legal disputes, the judges largely consult them. Thus, it is mandatory to know them and take them into account in clinical practice. Conversely, the greater restrictiveness of the COCIS can be explained by the fact that, in contrast to other states, only in Italy there is the figure of the sports physician who is responsible for the certificate of fitness for competitive sports. The other guidelines mentioned represent only a tool through which the cardiologist can recommend or not a certain sport activity based on the subject’s heart disease.

We believe that it is correct to stratify the risk of SCD in any category of athlete, whether amateur or competitive, recognizing the need for a certificate of fitness for competitive ones (to be drawn up by consulting protocols such as the COCIS) and the good practice of screening for all the other categories, looking for cardiopathies/channelopathies, as prompted by American or European guidelines.

Some diseases can be promptly anticipated by practicing primary prevention or can be promptly treated in such a way as to positively alter their courses, for example, by practicing secondary prevention. In particular, pre-participation screening represents the possibility to monitor athletes at risk of SCD and therefore stop their participation in competitions. This screening is not massively carried out for athletes but follows its own rules according to the countries and the type of sporting activity. Sudden death in athletes is a rare phenomenon (each year, 1–3/100.000 competitive athletes aged <35 years worldwide) [1,2,3], and it is not clear whether mass screening is cost-effective. In Italy, screening is performed by law for competitive sports and recently also for non-competitive ones [15,16]. In contrast, screening is not mandatory in the United States, by a principle of *“self-determination”* according to which anyone can play sports even if this may involve a risk of SCD. However, the ECG, a simple, short, easily accessible, and inexpensive exam, is sufficient in most cases to obtain clues about the presence of a heart disease or a channelopathy that involves a risk of SCD during sports. As early as 1997, Fuller et al. demonstrated the greatest efficacy of ECG rather than cardiological history and inspection/auscultation during a screening of 5615 high-school athletes for SCD risk [30]. Pelliccia et al. have proven that 12-lead ECG seems efficient in identifying young athletes with hypertrophic cardiomyopathy, leading to their timely disqualification from competitive sports [31]. In line with this, Cases 1, 2, and 3 are examples of how a careful and correct reading of the ECG could have led to a further diagnosis and maybe even could have helped avoid cardiac arrest. Therefore, it would be advisable for anyone who approaches sports, even as a hobby, to perform an ECG. Access must be free and strongly encouraged by information campaigns, and it would be of great value if it were offered free of charge in view of a spreading prevention. In light of a screening perspective, automatic ECG evaluation using artificial intelligence algorithms may be a promising tool [32]. Furthermore, attention must be paid to the correct execution of the 12-lead resting ECG: standard positioning and filters of the leads and no artifacts are not negligible to have a high-quality ECG and to be able to evaluate even subtle anomalies (e.g., QRS notching or slurring, mild delta or epsilon waves, small changes in J-ST segment or QT(c) duration, etc.).

Therefore, worldwide there is a variable percentage of subjects who play sports activities without the awareness of being suitable or not. On the other hand, as already stated, it is impossible to widely extend the habit of screening or a sports fitness visit to anyone and for any type of activity. Very often, sports are free and carried out outside sports infrastructures; for example, some people play them during free time. At the same time, amateur sports activities have no lower risk than competitive ones: we know for sure that most sudden deaths occur during amateur sports, which are often practiced outdoors and even in the most remote and isolated places [33]. Hence, there is an important need for greater availability of AEDs and people who are ready to practice basic life support–defibrillation (BLS–D), thus aiming for a scenario that can be defined as *“widespread cardioprotection”*. The need for sports-related SCD secondary prevention also arises from the fact that pre-participation evaluation may fail to recognize subjects at risk of sports-related SCD. It is not trivial. Case 4 is an example of a subject with a pathological resting ECG who nevertheless showed no signs of structural heart disease or channelopathy on diagnostic tests (although performed retrospectively after the aborted SCD event). The case, once again, and together with the others presented, marks the importance of resting ECG evaluation in intercepting subjects potentially at risk (given the presence of ECG-graphic alterations worthy of further diagnostics). On the other hand, all of these cases invite us to reflect on the imperative need for AEDs and CPR to be available during sports activities.

Borjesson et al. surveyed the emergency cardiovascular care at major sporting arenas in Europe, questioning the emergency planning, availability of AEDs, emergency personnel, and distance to the nearest hospital. Only 64% of major soccer arenas had a written medical action plan for cardiac arrest, and 72% had on-site AEDs. In addition, approximately 25% of the arenas situated longer than 10 min from the nearest hospital did not have an on-site AED [33]. These findings led to the development of European consensus recommendations on cardiovascular safety in sports arenas [17,18].

Moreover, what if cardiac arrest occurs outside a *“cardioprotected”* center?

To gain *“widespread cardioprotection”*, we need to increase the number of people who are able to recognize cardiac arrest and who can start CPR maneuvers. Most cardiac arrests occur in the presence of witnesses who could act as potential rescuers. In Europe, the average percentage of arrests witnessed is 66%. If, in Europe, witnesses intervened in all of these cases, instead of less than half, they could save about 100,000 people a year (data from **EuReCa One Registry**) [34].

Awareness and education are the keywords to spread the skills of BLS–D among people.

The media coverage of athletes suffering from cardiac arrest during competitions plays in favor of raising awareness of the problem. In addition, various countries promote awareness campaigns on the issue. For example, in Italy, since 2013, the Italian Resuscitation Council (IRC) has carried out “**Viva! La settimana per la rianimazione cardiopolmonare**” **(or “Viva! The week for cardiopulmonary resuscitation”)**; and since 2012, the European Parliament has promoted **October 16** as the day for CPR. Later, this date gained worldwide recognition with the **“World Restart a Heart Day”.**

Awareness and education are crucial, particularly in some specific contexts such as school and driving license [35]. If the correct information for overcoming motivational barriers (such as unawareness of the functioning of AEDs and the benefits of early CPR, reassurance about the personal and legal consequences of the intervention, etc.) are offered to start from school age, it is more likely that they will become familiar and helping will be perceived as a shared and desirable attitude [36]. It is important to point out how the compulsory training increases the number of trained individuals: a research in the USA highlights that the number of people trained to perform CPR maneuvers was higher in states where there is a law that makes CPR training mandatory, especially at school, at an appropriate age [37]. In this view, incentivizing training in BLS–D at school, at the maturity age or upon receiving a driving license, has a key role in the education of as many people as possible.

To summarize, many victims of cardiac arrest die because they do not receive the necessary help in time.

To increase the number of survivors (currently very low), it is necessary to increase the number of potential rescuers who are able to recognize cardiac arrest, at least starting chest compressions and applying an AED as soon as possible. In addition, the dissemination of AEDs is essential for this purpose, as well as the promotion of their use not only by trained people. Capucci et al. showed that allowing volunteers not trained in BLS–D to use AEDs tripled the survival rate of out-of-hospital SCD **(Piacenza Progetto Vita (or Piacenza Life Project)]** [38]. This aim requires equipping the AEDs stations with instructions necessary for their correct use and/or giving volunteer rescuers the opportunity to interact with emergency responders (e.g., 118 or 911) through calls or video-calls to receive assistance in real time.

Greater accessibility to AEDs can be achieved not only by increasing their number by placing them in “hot” fixed places such as squares, schools, and public offices but also in private buildings (both business and residential) in such a way as to have a homogeneous density and distribution proportional to the local population. Tax incentives from the state to individuals for AEDs purchases are of great importance. An interesting possibility is the use of medical drones to deliver AEDs in the place where cardiac arrest occurred [39]. In a lot of countries, the AEDs availability in sports infrastructures and during competitions is required by law [17,18], but it should be put into practice in all countries.

All the AEDs available in any territory must be registered, and a list of them can be consulted in real time by the rescuers. In this regard, APPs for smartphones have been developed for the geolocation of AEDs. For example, the **DAE RespondER** app, which is promoted by the Italian region Emilia-Romagna, was revealed to be very useful [40].

The Italian city Piacenza has become an example and a model thanks to the “**Progetto Vita**” (**or “Life Project”)**. It is the most cardio-protected city in Europe, with nearly 1000 AEDs distributed in the city (1 AED per 327 inhabitants) and an out-of-hospital cardiac arrest mortality rate four times lower than the average (online available at: https://www.defibrillatori.info/piacenza-la-citta-del-cuore/ from 3 December 2020).

The compilation of cardiac arrest records (with data collected from witnesses, rescuers, emergency services, pathologists, etc.) is a very useful tool to improve rescue measures. For this purpose, two registers, **EuReCa One** (closed) and **EuReCa Two** (ongoing), have been made.

Finally, we should to raise awareness toward behavioral follow-up for the prevention of drug abuse among athletes that can lead to heart problems or SCD after sport. In fact, in 2014, Maron et al. pointed out that, among young sports people, SCD occurs more frequently for causes such as suicide and drugs than for established cardiovascular causes [41].

## 7. Conclusions

Playing sports is a good habit but can be dangerous at all ages in the case of known or unknown cardiac pathologies. Periodic medical examinations are mandatory for athletes, but the importance of primary prevention has a limitation due to the difficulty to extend a mass screening. In a recent editorial, M. Ackerman advocates for the proactive identification for athletes at risk for SCD: not to “screen, identify, and disqualify” but to “screen, identify, risk stratify, and treat” [28]. However, screening is not enough to reduce sports-related SCD, and so we must also promote secondary prevention, not only creating *“cardioprotected”* infrastructures but also increasing the broad availability of AEDs and people trained in BLS–D.

## Data Availability

The data that support the findings of this article are available from the corresponding author, [S.R.], upon reasonable request.
